# Peripheral retinal nonperfusion using widefield imaging with von Hippel-Lindau disease

**DOI:** 10.1186/s40942-018-0139-6

**Published:** 2018-10-03

**Authors:** Jose S. Pulido, Lauren A. Dalvin, Timothy W. Olsen, Fukutaro Mano, Michael Yu, Carol L. Shields

**Affiliations:** 1 Department of Ophthalmology, Mayo Clinic, 200 First Street, SW, Rochester, MN 55905 USA; 2 Department of Molecular Medicine, Mayo Clinic, 200 First Street, SW, Rochester, MN 55905 USA; 3 Ocular Oncology Service, Wills Eye Hospital, Thomas Jefferson University, 840 Walnut Street, 14th Floor, Philadelphia, PA 19107 USA

**Keywords:** Eye, Tumor, Hemangioblastoma, Fluorescein angiography, Nonperfusion, von Hippel-Lindau

## Abstract

**Background:**

To describe a case of von Hippel-Lindau disease with peripheral retinal nonperfusion.

**Case presentation:**

A 66-year-old female with known cerebellar and midbrain hemangioblastomas was evaluated for a retinal hemangioblastoma in the right eye. She underwent widefield fluorescein angiography, which showed hyperfluorescence localized to the hemangioblastoma surrounded by peripheral retinal nonperfusion in the same quadrant.

**Conclusions:**

Further widefield imaging studies are required to determine if peripheral retinal nonperfusion is a common finding in von Hippel-Lindau disease.

## Background

Prior studies have shown that patients with von Hippel-Lindau (VHL) disease can develop not only retinal hemangioblastomas but also retinal neovascularization [1, 2]. The etiology of retinal neovascularization in VHL is unknown. We describe widefield fluorescein angiography features of a patient with VHL.

## Case presentation

A 66-year-old female with known VHL disease was referred for ocular evaluation. Systemically, she had cerebellar and midbrain hemangioblastomas. She had prior trauma to her left eye 40 years earlier for which she had undergone evisceration. She had open-angle glaucoma in the right eye for which she was using latanoprost and timolol maleate once daily.

**Fig. 1:**
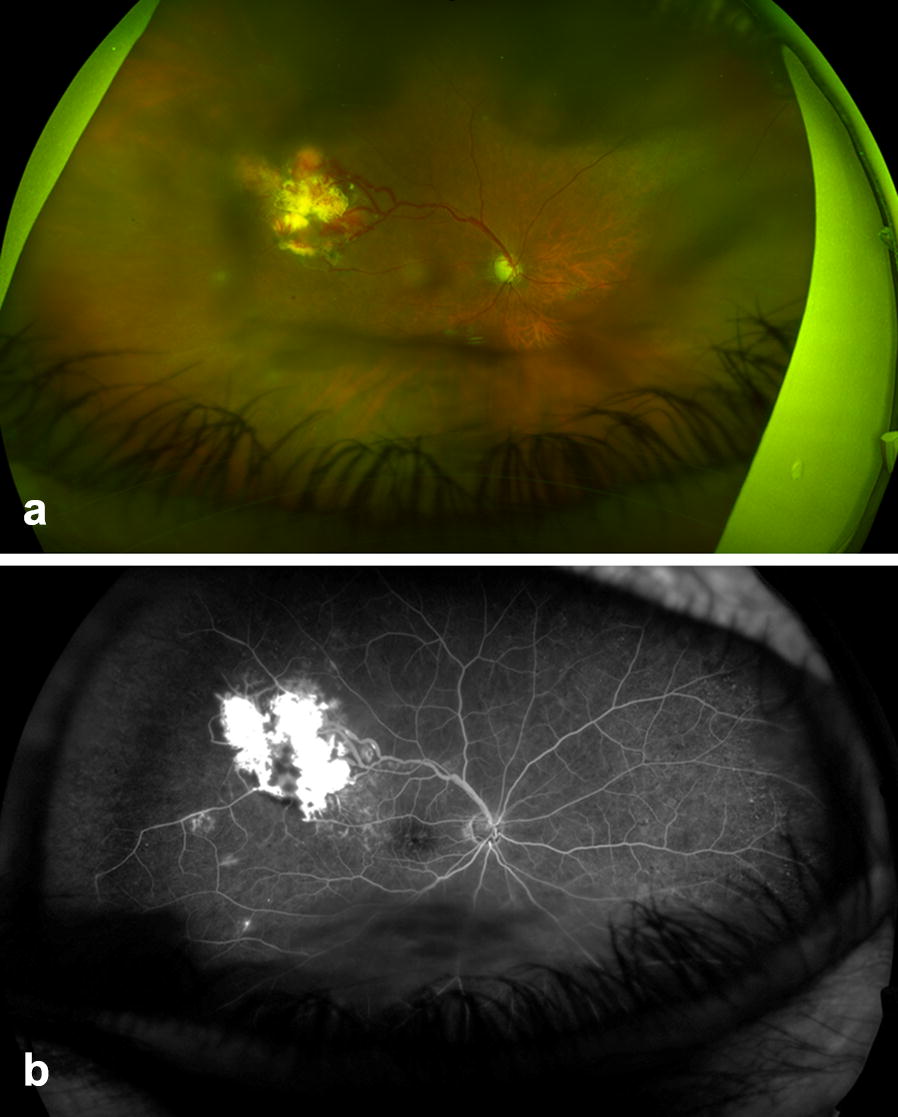
**a** Widefield Optos imaging of the right eye of a patient with von Hippel-Lindau disease demonstrates a retinal hemangioblastoma in the superotemporal quadrant with associated dilated feeding and draining vessels. **b** Widefield fluorescein angiography of the right eye reveals fluorescein uptake and leakage from the hemangioblastoma with peripheral retinal nonperfusion in the superotemporal quadrant anterior to the tumor

**Fig. 2:**
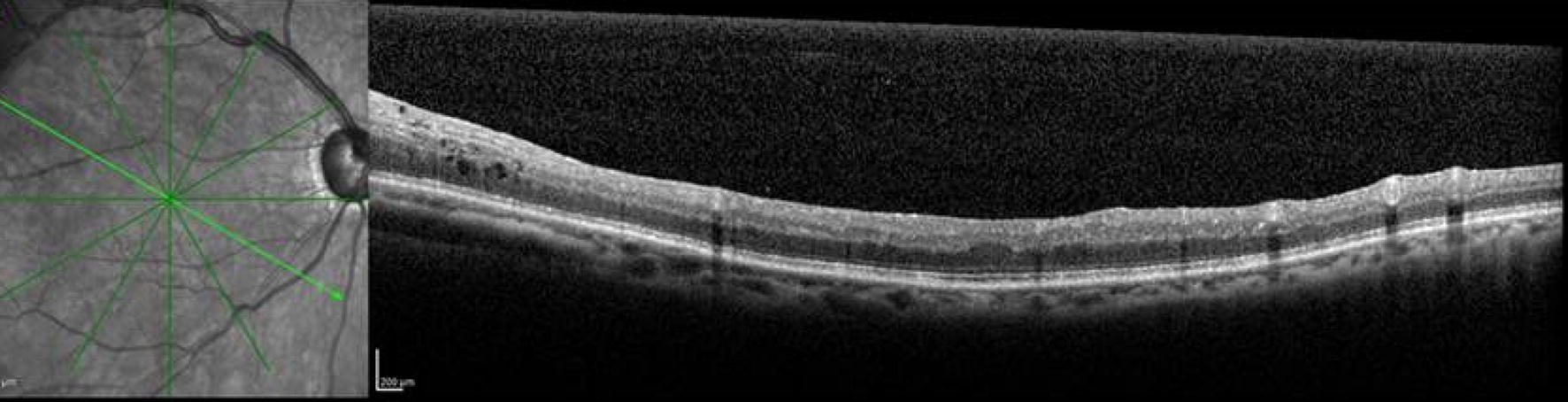
Optical coherence tomography of the right eye reveals intraretinal fluid extending from the hemangioblastoma towards the temporal macula

On presentation, visual acuity was 20/60 in the right eye with intraocular pressure of 15 mmHg. The anterior segment examination of the right eye was unremarkable. On funduscopic examination, a VHL-related retinal hemangioblastoma was seen superotemporal to the macula associated with dilated feeding and draining vessels (Fig. 1). There was no peripheral retinal detachment, but there was intraretinal fluid extending from the hemangioblastoma towards the temporal macula seen by optical coherence tomography (Fig. 2).

Widefield fluorescein angiography was performed, which showed fluorescein uptake and leakage from the hemangioblastoma and peripheral retinal nonperfusion anterior to the tumor in the superotemporal quadrant. Treatment of the hemangioblastoma and peripheral retinal nonperfusion was recommended; but because this was her only eye, the patient preferred close observation with monitoring for progression.

## Discussion and conclusions

With widefield Optos fluorescein angiography, we have demonstrated a case of retinal nonperfusion peripheral to a VHL-associated hemangioblastoma. The underlying pathophysiology of peripheral retinal nonperfusion in VHL is unknown. We hypothesize that the hemangioblastoma could be causing a vascular steal syndrome from the peripheral retina, which subsequently causes the nonperfusion.

This peripheral steal phenomenon could occur in other VHL hemangioblastomas. However, we suspect that peripheral retinal nonperfusion has previously gone unnoticed due to a lack of peripheral fluorescein angiography imaging in these cases. This finding might help explain why we and others have seen retinal neovascularization in the past. Larger hemangioblastomas might exhibit greater steal and, therefore, cause further nonperfusion with ischemia and the development of neovascularization. Nonperfusion related to vascular steal likely begins in the retina immediately anterior to the tumor. However, nonperfusion could expand to other quadrants over time. In our patient, nonperfusion was localized to the superotemporal quadrant. Long term follow up studies would be required to assess change in area of nonperfusion over time.

VHL is caused by a tumor suppressor gene mutation. The VHL protein ubiquitinates hypoxia inducible factor-1alpha (HIF-1α), allowing subsequent trafficking of HIF-1α to the proteasome for breakdown of the HIF-1α protein [3]. In the absence of HIF-1α ubiquitination, HIF-1α forms a heterodimer with HIF-1β, resulting in elevated transcription of HIF target genes in all cells in the body [3]. Thus, VHL-associated hemangioblastomas have high levels of heterodimeric HIF [4]. Elevated levels of HIF cause a cascade of events, including upregulation of vascular endothelial growth factor (VEGF) [4]. Subsequent development of peripheral retinal nonperfusion in a patient with VHL likely causes further upregulation of HIF heterodimers and VEGF, which could be the impetus for retinal neovascularization. We suspect that prophylactic treatment to areas of peripheral retinal nonperfusion related to retinal hemangioblastoma could decrease levels of VEGF and prevent development of vision-threatening neovascularization.

Larger studies are needed to investigate the prevalence of peripheral retinal nonperfusion in VHL patients and to determine whether nonperfusion correlates with hemangioblastoma size and development of retinal neovascularization.

## Abbreviations

VHL: von Hippel-Lindau
HIF: hypoxia inducible factor
VEGF: vascular endothelial growth factor
